# The effect of comprehensive assessment and multi-disciplinary management for the geriatric and frail patient: A multi-center, randomized, parallel controlled trial

**DOI:** 10.1097/MD.0000000000022873

**Published:** 2020-11-13

**Authors:** Simin Yao, Peipei Zheng, Liwei Ji, Zhao Ma, Lijuan Wang, Linlin Qiao, Yuhao Wan, Ning Sun, Yao Luo, Jiefu Yang, Hua Wang

**Affiliations:** a Department of Cardiology, Beijing Hospital, National Center of Gerontology; Institute of Geriatric Medicine, Chinese Academy of Medical Sciences; b Peking University Fifth School of Clinical Medicine, Dong Dan, Beijing; c Department of Pharmacy; d Department of Rehabilitation; e Department of Nutriology; f Department of TCM, Beijing Hospital, National Center of Gerontology, Institute of Geriatric Medicine, Chinese Academy of Medical Sciences, PR China.

**Keywords:** frailty, comprehensive geriatric assessment, multi-disciplinary management, rehabilitation exercise, diet adjustment, multi-drug evaluation, traditional Chinese medicine, patient education

## Abstract

**Background:**

A comprehensive geriatric assessment (CGA) of elderly patients is useful for detecting the patients vulnerabilities. Exercise and early rehabilitation, nutritional intervention, traditional Chinese medicine (TCM), standardized medication guidance, and patient education can, separately, improve and even reverse the physical frailty status. However, the effect of combining a CGA and multi-disciplinary management on frailty in elderly patients remains unclear. The present study assessed the effects of a CGA and multi-disciplinary management on elderly patients with frailty in China.

**Methods:**

In this study, 320 in patients with frailty ≥70 years old will be randomly divided into an intervention group and a control group. The intervention group will be given routine management, a CGA and multi-disciplinary management involving rehabilitation exercise, diet adjustment, multi-drug evaluation, acupoint massage in TCM and patient education for 12 months, and the control group will be followed up with routine management for basic diseases. The primary outcomes are the Fried phenotype and short physical performance battery (SPPB). The secondary outcomes are the clinical frailty scale (CFS), non-elective hospital readmission, basic activities of daily living (BADL), 5–level European quality of life 5 dimensions index (EQ-5D), nutrition risk screening-2002 (NRS-2002), medical insurance expenses, fall events, and all-cause mortality. In addition, a cost-effectiveness study will be carried out.

**Discussion:**

This paper outlines the protocol for a randomized, single-blind, parallel multi-center clinical study. This protocol, if beneficial, will demonstrate the interaction of various intervention strategies, will help improve elderly frailty patients, and will be useful for clinicians, nurses, policymakers, public health authorities, and the general population.

**Trial registration:**

Chinese Clinical Trials Register, ChiCTR1900022623. Registered on April 19, 2019, http://www.chictr.org.cn/showproj.aspx?proj=38141.

## 1 Introduction

Aging is an important public health issue all over the world. Frailty is a nonspecific state characterized by decreasing muscle strength, endurance and physiological reserves that may occur as the result of a variety of diseases and clinical conditions, resulting in increased vulnerability and a decreased anti-stress ability in elderly individuals.^[1]^ The prevalence rate of frailty increases with age, being around 10% in people ≥65 years old and 25% to 50% in people >85 years old.^[2]^ In addition, the prevalence rate of frailty is higher in medical institutions than in community-residing elderly.^[3–5]^ It seriously affects the function and quality of life of the elderly and poses a great threat to health life expectancy. Compared with non-frail elderly, frail elderly were shown to be more likely to suffer negative clinical events and require substantial medical resources.^[6]^ Several large epidemiological studies showed that frailty was strongly related to a poor prognosis of individuals, and it was an independent predictor of falls,^[1]^ deterioration of the function,^[1]^ decreased mobility,^[7]^ hospitalization, and death.^[8]^

Intervention studies on frailty around the world are still in their infancy.^[9–11]^ A few randomized controlled studies have found that multidisciplinary intervention can effectively treat and improve frailty in elderly patients.^[12–14]^ Exercise and early rehabilitation protocols applied during hospitalization can prevent functional and cognitive decline in older patients and were shown to be associated with a reduced length of stay and lower costs.^[15]^ Combined aerobic and resistance training can improve interventions in physical function and help reduce frailty.^[16]^ Exercise is likely the best therapy for reversing the frailty status, including aerobic, resistance, flexibility, and balance training components. More than 30% of medical inpatients are at an increased risk of malnutrition, a condition that is strongly associated with increased mortality and morbidity, medical resource use and a reduced functional ability.^[17]^ While nutritional intervention can improve a persons physical condition, traditional Chinese medicine (TCM) therapies appear to be effective for treating fibromyalgia, insomnia and constipation,^[18–20]^ and acupoint massage is an effective intervention for maintaining the cognitive function among older adults.^[21]^ Insomnia and constipation are common diseases in the elderly, so effective and simple acupoint massage may improve the symptoms of insomnia and constipation in daily life. Frail individuals tend to be affected by polypharmacy and are at an increased risk of adverse drug events. Standardized medication guidance is essential for improving the prognosis of elderly individuals with frailty.

A comprehensive geriatric assessment (CGA) of elderly patients is useful for detecting patients vulnerabilities,^[22]^ identifying elderly patients at high risk for mortality and poor recovery after surgery,^[23]^ and predicting outcomes independent of performance status among elderly patients.^[24]^ Multi-disciplinary intervention according to the CGA findings has been shown to improve the activity, anxiety and depression status, fall events, readmission rate, and quality of life score to a certain extent.^[25–27]^ Therefore, combining a CGA and multi-disciplinary intervention may be an effective means of improving frailty in elderly patients.

Hospitalization may lead to a decline in the function of elderly patients, and many elderly patients are unable to fully recover their physical function after hospitalization, which increases the risk of frailty, disability, dependence, re-hospitalization and death.^[28,29]^ This point therefore merits a thorough look.

In the present study, frailty will be measured using the definition of frailty syndrome established by Fried et al,^[1]^ and patients in the intervention group will undergo the CGA during hospitalization and start multi-disciplinary intervention that will last 12 months. If this study proves that the multi-disciplinary management model can improve the symptoms and prognosis of frail elderly patients, it will help improve the medical status of elderly frailty patients, save social public medical resources and provide assistance for the development of medical policies.^[30,31]^

## 2 Methods

### 2.1 Study design and setting

This is a multicenter, randomized, parallel controlled study. The study has been designed to determine the effect of a CGA and multi-disciplinary management on patients ≥70 years old with frailty in China.

The primary outcomes are the Fried phenotype,^[1]^ which presenting at least 3 of the following: unintentional weight loss, self-reported exhaustion, weakness, slow walking speed and low physical activity; and the short physical performance battery (SPPB), scored from 1 to 12 based on three tests (balance tests, gait speed and 5-sit-to-stand test; low score 0–6, medium score 7–9, high score 10–12).^[32]^ The secondary outcomes are the clinical frailty scale (CFS), which uses pictographs and clinical descriptions to help clinicians stratify elder adults on a 9-point scale (1 being very fit and 9 being terminally ill);^[33]^ non-elective hospital readmission,^[13]^ which is considered any type of emergency readmission; disability, which is assessed by basic activities of daily living (BADL: total score 6);^[34]^ health-related quality of life, which is evaluated by the 5–level European Quality of Life 5 Dimensions index (EQ-5D: index values range from 0 to 1, with higher scores indicating a better quality of life);^[12]^ medical insurance expenses; fall events; nutrition risk screening-2002 (NRS-2002: the total score ranges from 0 to 7, and a total score ≥3 indicates malnourishment),^[35]^ and all-cause mortality.^[36]^ A standard multiplication model will be used to estimate the quality adjusted life years (QALYs), which are calculated based on the EQ-5D or BADL.^[37]^ The enrollment, intervention allocation, follow-up and data analysis will be conducted according to the Standard Protocol Items: Recommendations for Interventional Trials (SPIRIT) statement.^[38,39]^

### 2.2 Eligibility criteria

Inclusion criteria for recruitment: hospitalized and ≥70 years old; have a Fried phenotype ≥3; are living in Beijing for at least 1 year and able to participate in the follow-up of the study; are able to volunteer to participate and sign informed consent.

Exclusion criteria for recruitment: currently participating in other similar clinical trials; have been treated with regular (≥3 days per week) traditional Chinese medicine (TCM) or acupuncture or have received regular (≥3 days per week) nutritional supplements or received treatment at a rehabilitation facility within the last 3 months; have exercised regularly at moderate intensity over the past 3 months (maximum heart rate of 50% × (220 – age) during exercise);^[40]^ are unable to complete the SPPB (main result of the study); are unable to participate in and stick to exercise alone; will be residing in a hospital or care home, including community specialist nursing facilities, rehabilitation centers and intermediate care facilities in the coming year; have dementia or severe cognitive impairment; have other severe concomitant conditions impair the ability of the patient; have end-stage disease with a life expectancy <12 months; have any other conditions or events that researchers or therapists consider to be excluded.

### 2.3 Termination and withdrawal criteria

In the course of the study, if the researchers consider that continuing to participate in the study will lead to adverse outcomes, then the subjects participation will be terminated and they will be asked to withdraw from the study. The subjects are also free to request withdrawal at any time.

### 2.4 Sample size

The sample size will be determined according to the difference test formula of the mean comparison between 2 groups (Power = 80%, *P* = .05). In this study, the Fried phenotype and SPPB will be taken as the main endpoints. According to previous studies, a sample of 250 (125 per group) is required to detect a 33% increment in the mean number of frailty criteria (from 1.2 to 1.6),^[13]^ a sample of 256 (128 per group) is required to detect a 41% between-group difference in the average reduction in the number of frailty criteria (from 0.41 to 0.8),^[12]^ and a sample of 202 (101 per group) is required to detect a 1.44-points between-group difference in average increment on the 12-point SPPB scale (from −0.98 to 0.52).^[12]^ With an attrition rate of 15%, at least 320 participants (160 per group) will be recruited.

### 2.5 Research method and procedures

#### 2.5.1 Outline

**Figure 1:**
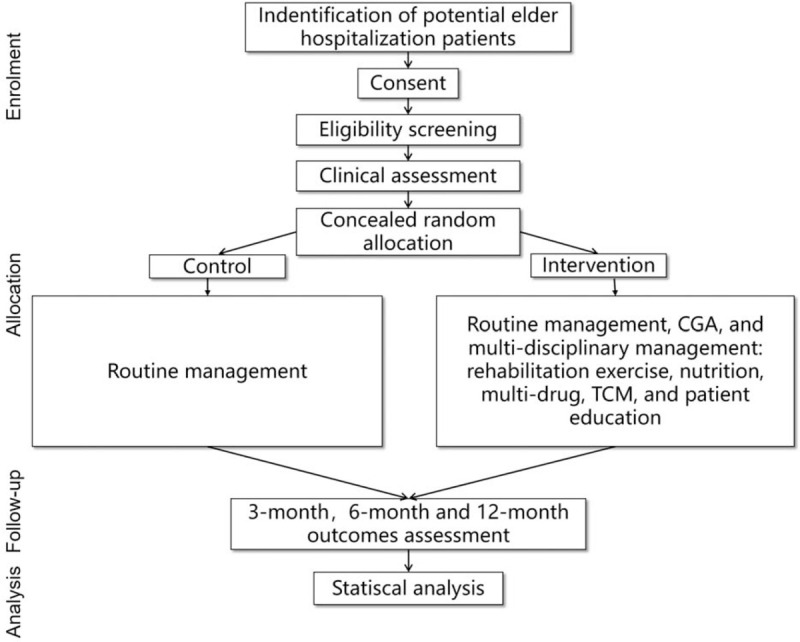
Overview of the participant flow for assessing the effect of a CGA and multi-disciplinary management on geriatric frail patients in China. CGA: comprehensive geriatric assessment; TCM = traditional Chinese medicine.

In this study, 320 elderly participants ≥70 years old will be randomly divided into the intervention group and control group. The intervention group will be given a routine diagnosis and treatment, a CGA and multi-disciplinary management for 12 months, and the control group will be followed up for 12 months with routine management for basic diseases. A flowchart of the trial is shown in Figure 1.

#### 2.5.2 Recruitment process

A schematic overview of the outcomes, measures and timeline is shown in Table 1. The researchers will recruit participants who meet the inclusion criteria and do not meet the exclusion criteria from Beijing Hospital, PLA General Hospital and Tsinghua Changgeng Hospital.

**Table 1:** Schedule of enrollment, interventions, and assessments.

	Visit 1	Visit 2	Visit 3	Visit 4
Time (months ± days)	**0** ± **5**	**3** ± **14**	**6** ± **30**	**12** ± **30**
Consent	√			
Inclusion/Exclusion Criteria	√			
Randomization	√			
Clinical Evaluation				
Demographic data	√			
Medical history	√			
Physical examination	√	√	√	√
Related auxiliary examination (±days), including routine blood test (±7), blood biochemical (±7) D-dimer (±7), NT-pro-BNP (±7), 25-OH-VD3 (±7), hsCRP (±7), thyroid function (±30), echocardiogram (±30).	√		√	√
CGA^∗^	√	√	√	√
Multi-disciplinary Management^∗^	√	√	√	√
Primary Outcomes		√	√	√
Secondary Outcomes		√	√	√
Adverse Event	√	√	√	√

All participants will receive a clinical evaluation by a nurse. Data on sociodemographic characteristics will be collected, including age, sex, marital status, educational level, smoking and alcohol consumption, and living arrangement. The health condition will be assessed by recording the number of comorbidities, fall occurrences in the past, sleeping quality (Pittsburgh sleep quality index [PSQI]),^[41]^ functional status (BADL, instrumental activity of daily living [IADL]),^[42]^ simple nutritional status (diet, appetite, and calf girth), physical activity or exercise, results of a special physical examination, psychosocial components (mini-mental state examination [MMSE],^[43]^ hospital anxiety and depression scale [HADS-A]),^[44]^ geriatric depression scale [GDS-5 items]),^[45]^ and related auxiliary examination (Table 1).

#### 2.5.3 Randomization and blinding

A total of 320 participants will be randomly assigned by a central computer randomization software program; 160 participants in the intervention group and 160 participants in the control group. The randomization sequence is automatically generated when the researchers complete the clinical evaluation and input the electronic data capture system (EDC), and artificial intervention is impossible.

This study is a single-blind trial design. Considering that the study needs the cooperation of participants and multi-disciplinary intervention teams, it is not possible to blind participants and staff administering interventions to group allocation. However, the staff performing the clinical evaluations, outcome assessors and data analysts will be blinded to the intervention group assignment. Assessors will not be involved in intervention activities.

#### 2.5.4 Bias control

The endpoint events of this study are all objective indicators. Researchers will quantify these objective indicators and establish unified and clear criteria. The assessors will receive unified and standardized training before investigation. Through brief telephone reviews and record books, a good trust relationship with participants will help reduce selection bias and increase compliance.

### 2.6 Intervention

Participants in the intervention group will receive a CGA and receive 12 months multi-disciplinary management. Interventions will be carried out by a multi-disciplinary team (including physicians, nurses, nutritionists, rehabilitation physicians, pharmacists, TCM physicians). A brief CGA will be carried out by nurses during the three days of hospitalization, including evaluations of the following data: hearing impairment, visual impairment, masticatory disorder, risk of fall and imbalance, history of falls, urinary incontinence and fecal incontinence, chronic constipation, and chronic pain. The specialized CGA and interventions are as follows:

1. Nutrition aspect
  Assessment: Age, grip strength, daily physical activity, NRS-2002 score, clinical diagnosis (especially sarcopenia), laboratory results, human body composition analysis (Inbody 720), dietary survey.
  Advice: A reasonable dietary plan will be formulated by the nutritionist.
2. Rehabilitation
  Assessment: SPPB, physical fitness tests,^[46]^ including a 30-second sit-to-stand test^[47–49]^ to assess lower body strength, a 30-second arm curl test^[50]^ to assess upper body strength, a 2-minutes step test^[51]^ to assess the cardiopulmonary function and aerobic endurance, a chair sit-and-reach test^[52,53]^ to assess lower body flexibility, a back scratch test^[54]^ to assess upper body flexibility, and a time-up-and-go test (TUG)^[53,54]^ to evaluate dynamic balance and agility.
  Advice: A set of rehabilitation exercises will be taught, including breath adjustment, posture adjustment, guidance for contracting abdominal muscles and pelvic floor muscles, head and neck stretching, back scratching, sitting forward stretching, sitting elastic belt assisted upper limb movement, heel lifting and stepping. Each participant will be given a sports manual with exercise instructions and safety information. During the period of hospitalization, all training sessions will be carefully supervised by a rehabilitation physician experienced in therapeutic training. Participants will perform 3 to 20 repetitions for each exercise and 3 to 5 times per week for 12 months, and accurately record the frequency, time and feelings concerning exercise each week, gradually increasing to the maximum tolerance and safety intensity. Participants will also be encouraged to exercise on their own and walk for a few minutes each day.
3. Multi-drug evaluation
  Assessment: Medication appropriateness index (MAI),^[55]^ review of the type, measurement and quantity of drugs used by patients during hospitalization.
  Advice: Pharmacists will participate in the administration of drugs to the participants, potentially harmful drugs or doses will be revised and patients will receive instructions concerning medication when discharged.
4. TCM
  Assessment: TCM physicians will comprehensively and systematically assess the participants based on the TCM theory of syndrome differentiation, including a TCM tongue diagnosis and pulse diagnosis.
  Advice: TCM physicians will deliver guidance on performing acupoint massage related to constipation and insomnia, including Zhigou, Zusanli, Dachangshu, Neiguan, Shenmen, and Sanyinchiao. Each participant will be instructed on 0 to 4 related acupoints, and each acupoint will be massaged for 5 minutes every day.
5. Patient education
  Nurses will issue a patient education manual to each participant, covering the basic introduction of frailty, fall prevention education, first-aid knowledge and cardiovascular medicine guidance. All participants in both groups will undergo routine management.

### 2.7 Follow-up

Data will be collected from all participants at 4 points throughout the study: before the intervention and 3, 6, and 12 months after the enrollment. Brief telephone reviews will be conducted approximately 2 to 3 times per month for all participants to promote adherence to the intervention. Participants in both groups will receive at least 12 months of telephone follow-up after the completion of the assessment or intervention in order to assess the durability and sustainability of any influence identified once the intervention stops.

Participants in the intervention group will be defined as treatment-compliant if they complete at least 50% of the planned rehabilitation exercises and/or 50% of their planned nutrition guidance program and 50% of the acupoint massage prescribed by TCM within 12 months. Participants will be required to make records in the debilitating record book. The individual dietary plan, rehabilitation exercise instruction with pictures and safety information, medication instruction, diagram of acupoint massage and patient education manual will also be represented in the record book in order to increase patients compliance. Researchers will assess the actual completion through the above requirements at the end of the intervention.

### 2.8 Statistical management and analyses

Statistical management: EDC will be established for standardized management. All data of the participants can be traced back to the source. Peking university clinical research institute will mainly participate in the process of establishing EDC, data statistics and quality control of the trial. Full-time supervisors will examine the whole process of the trial. We will regularly publish the research progress and resolve issues in the research.

Statistical analyses: We will conduct an intention-to-treat (ITT) analysis for the primary analysis, including primary and secondary outcomes, with a per-protocol (PP) analysis used for the sensitivity analysis. The ITT population is defined as all randomized participants in the study, and the PP population is defined as all randomized participants who were actually compliant with their treatment. The participants clinical features will be compared between the intervention and control groups. Continuous variables will be expressed as the mean ± standard deviation in a normal distribution and median and interquartile range in a non-normal distribution, and categorical variables will be expressed as numbers and percentages. Baseline characteristics will be compared by χ^2^ tests or Fishers exact tests for categorical variables and *t*-tests or Wilcoxons rank sum tests for continuous variables. A covariance analysis will be used to analyze the Fried phenotype, SPPB and modified means of the 2 groups, taking the central effect of the test into account, and the 95% confidence interval (CI) of the difference between the 2 groups will be calculated. Only when the above 2 main indicators satisfy the statistical hypothesis (superior effect), can the hypothesis be considered valid. For time-event data, the Kaplan–Meier method will be used to estimate death, non-elective hospital readmission and fall events. A log rank test will be performed for the 2 groups. A Cox proportional-hazard regression model will be used to calculate the effect of potential risk factors on the events. We will summarize the number of enrollments and completion of each center, list the drop-outs, compare the total drop-out rate and list the reasons for termination in detail and compare the characteristics between those with missing outcome data and those who have completed follow-up. The safety evaluation table will be included to describe the number of adverse events and the incidence of adverse events, serious adverse events, adverse reactions and serious adverse reactions in the two groups. Statistical significance will be set at a 2-sided *P* value <.05.

A cost-effectiveness analysis will also be performed to determine whether or not CGA and multi-disciplinary management interventions are cost effective relative to no intervention. The costs of outpatient visits, readmissions and delivering the intervention; the type of hospital admission and number of hospitalization; and the length of stay in the hospital within 12 months will be estimated from the perspective of the medical insurance institutions. The number of assessors and interveners, working hours, labor costs, equipment costs and consultation expenses will be obtained. A standard multiplication model will be used to estimate the QALYs, calculated based on the EQ-5D or BADL for each individual using the monthly interval.^[37]^ We will estimate life-years (LYs) using individual patient data by calculating the time to death from recruitment.^[37]^ Costs and QALYs or LYs will be summed as a decision model to estimate incremental cost-effectiveness ratios.

### 2.9 Adverse events and severe adverse events

#### 2.9.1 Adverse events: fractures associated with rehabilitation exercise

Severe adverse events: persistent or severe disability or dysfunction caused by rehabilitation exercise.

If adverse or severe adverse events occur, the relationship between them and the intervention will be assessed, the intensity and mode of intervention will be adjusted, the intervention will be suspended if necessary, and the participants will receive appropriate treatment. We will immediately provide a diagnosis and treatment and report the occurrence in a timely manner while compensating the participant according to the relevant laws and regulations of our country.

#### 2.9.2 Ethics and dissemination

This study was reviewed and approved by Ethics Committee of Beijing Hospital, China. (ID number: 2019BJYYEC-045–02). All participants will sign their informed consent according to the Declaration of Helsinki prior to data collection. The results of this trial will be presented at scientific conferences and published in peer-reviewed journals. The design and development of this study is patient-centered. Patients were not involved in the development or design of the study. The burden of the intervention will not be assessed by patients themselves. Patients or their guardians will receive a verbal and written summary of the tests and evaluation results, as well as the study results that are allowed to be disseminated to them.

## 3 Discussion

At present, there is still a lack of effective guidelines for the management of elderly frailty patients. As the most effective measure to improve physical fitness and muscle strength, rehabilitation exercise and reasonable diet have great heterogeneity. Moreover, the safety, cost-effectiveness, long-term sustainability and population acceptance ability of rehabilitation exercise or reasonable diet still need large randomized control tests or systematic reviews to verify. As a large nation of catering, the food culture of China is very pluralistic, but many old people in China have a deep-rooted food culture. Simple nutritional assessment and diet are particularly needed to guide the daily diet of the elderly. We hope that the nutrition intervention program designed in our study can be popularized. In addition, we will teach the appropriate content of standard rehabilitation exercise according to the results of physical fitness assessment, and recommend the best exercise intensity to the patients. Moreover, standardized medication guidance, simple acupoint massage on insomnia and constipation and patient education can be well performed by a team in clinical work. A remarkable characteristic of our study is the utilization of an interdisciplinary team (physicians, nurses, nutritionists, rehabilitation physicians, pharmacists, TCM physicians) that manages for inpatients in different clinical departments. Our result may be able to show that an individualized multi-component prescription for elderly frailty patients is plausible.

This paper outlines the protocol for a randomized, single-blind, parallel, multi-center clinical study to explore the prognosis of elderly frailty patients after CGA and multi-disciplinary management in China. The main purpose of this paper is to explore a diagnosis and treatment management mode for elderly frailty patients, rather than to evaluate the weight of a specific measure among a variety of interventions, since the effect of each measure has been clearly demonstrated in previous studies. If our hypotheses are correct, we can establish a set of systematic and standardized management mode. Compared with the control group, the cost-effectiveness analysis of CGA and multi-disciplinary management is the focus of our research, and also the key part of whether the research results can be popularized.

The main limitation to our study is that it takes a little long time to evaluate the particularly frailty elderly patients, but only an accurate CGA can better complete the intervention measures, and we can cooperate with all patients to complete the evaluation content when they are enrolled through strict inclusion-exclusion criteria and the assessment can also be conducted in several times, and for simple questions, we can also consult the patients family members or caregivers.

There is strong evidence to support CGA in a hospital setting in the Europe and North America, and randomized evidence from China would further strengthen the evidence base. The intervention being tested is CGA with the addition of TCM, if it is effective, it may further supplement the existing CGA. The study results will be extremely relevant to elderly patients. This protocol, if beneficial, will demonstrate the interaction of various intervention strategies, wherein a rehabilitation exercise, diet adjustment, multi-drug evaluation, acupoint massage in TCM and patient education are included, will help improve elderly frailty patients, and will be useful for clinicians, nurses, policymakers, public health authorities and the general population.

### 3.1 Trial status

At present, hospital and patient recruitment for this trial is ongoing. The first patient was included in June 2019 and recruitment is expected to be completed in December 2020. Protocol version number is 1.0, dated March 10, 2019.

## Acknowledgments

The authors thank Lingling Cui, Di Guo, Fei Li, Mengyan Sun, Xiaoya Zhang, Haifeng Wang for their support as assessors and interventionists, and thank the Peking university clinical research institute for supervising the whole process of the trial.

## Author contributions

HW and JY contributed in the conception of the idea for the study. SY and PZ contributed in the development of the protocol, organization and writing the manuscript. SY, PZ, LJ, ZM, LW, LQ, YW, NS, YL, JY are responsible for ongoing study procedures, data management and analyses. PZ was responsible for contributing to the calculation of the sample size. All of the authors read the draft, made contributions and approved the final manuscript.

**Conceptualization:** Simin Yao, Peipei Zheng, Jiefu Yang, Hua Wang.

**Formal analysis:** Peipei Zheng.

**Methodology:** Simin Yao, Peipei Zheng, Liwei Ji, Zhao Ma, Lijuan Wang, Linlin Qiao, Yuhao Wan, Ning Sun, Luo Yao, Hua Wang.

**Software:** Peipei Zheng.

**Supervision:** Jiefu Yang, Hua Wang.

**Writing – original draft:** Simin Yao, Peipei Zheng.

**Writing – review & editing:** Hua Wang.
